# Intracerebroventricular administration of the thyroid hormone analog TRIAC increases its brain content in the absence of MCT8

**DOI:** 10.1371/journal.pone.0226017

**Published:** 2019-12-06

**Authors:** Soledad Bárez-López, Carmen Grijota-Martínez, Xiao-Hui Liao, Samuel Refetoff, Ana Guadaño-Ferraz

**Affiliations:** 1 Department of Endocrine and Nervous System Pathophysiology, Instituto de Investigaciones Biomédicas Alberto Sols, Consejo Superior de Investigaciones Científicas (CSIC)-Universidad Autónoma de Madrid (UAM), Madrid, Spain; 2 Center for Biomedical Research on Rare Diseases (Ciberer), Instituto de Salud Carlos III, Madrid, Spain; 3 Department of Cell Biology, Faculty of Biology, Universidad Complutense de Madrid, Madrid, Spain; 4 Department of Medicine, The University of Chicago, Chicago, Illinois, United States of America; 5 Department of Pediatrics, The University of Chicago, Chicago, Illinois, United States of America; 6 Committee on Genetics, The University of Chicago, Chicago, Illinois, United States of America; University Claude Bernard Lyon 1, FRANCE

## Abstract

Patients lacking the thyroid hormone (TH) transporter MCT8 present abnormal serum levels of TH: low thyroxine and high triiodothyronine. They also have severe neurodevelopmental defects resulting from cerebral hypothyroidism, most likely due to impaired TH transport across the brain barriers. The use of TH analogs, such as triiodothyroacetic acid (TRIAC), that can potentially access the brain in the absence of MCT8 and restore at least a subset of cerebral TH actions could improve the neurological defects in these patients. We hypothesized that direct administration of TRIAC into the brain by intracerebroventricular delivery to mice lacking MCT8 could bypass the restriction at the brain barriers and mediate TH action without causing hypermetabolism. We found that intracerebroventricular administration of therapeutic doses of TRIAC does not increase further plasma triiodothyronine or further decrease plasma thyroxine levels and does not alter TH content in the cerebral cortex. Although TRIAC content increased in the brain, it did not induce TH-mediated actions on selected target genes. Our data suggest that intracerebroventricular delivery of TRIAC has the ability to target the brain in the absence of MCT8 and should be further investigated to address its potential therapeutic use in MCT8 deficiency.

## Introduction

Thyroid hormones (TH), 3,5,3’-triiodothyronine (T3) and thyroxine (T4) play an essential role in most tissues, including the developing and the adult CNS. Most actions of TH are mediated by the regulation of gene expression through binding of T3 to its nuclear receptors, alpha and beta [1]. Recent findings from several groups indicate that TH need transporter proteins to cross cellular membranes [2] among which is the monocarboxylate transporter 8 (MCT8), a TH-specific cell membrane transporter [3] that plays an essential role in TH function and action [1]. The gene encoding this transporter, *SLC16A2*, is located on the X chromosome, and inactivating mutations result in the Allan–Herndon–Dudley syndrome in males [4–6]. Patients present global developmental delay, severe intellectual disability, lack of speech and poor head control, as well as profound neuromotor impairments with central hypotonia, progressive spastic quadriplegia and dystonic movements [7]. Patients also present altered serum concentrations of TH with elevated T3, causing hyperthyroidism in peripheral tissues, low T4 and 3,5’,3’-triiodothyronine (reverse T3 or rT3), and normal or slightly elevated thyrotropin (TSH) [5, 6]. Currently there is much evidence to support that an impaired TH transport across the brain barriers (BBs; the blood-brain and the blood-cerebrospinal fluid barriers) is an important pathophysiological mechanism in MCT8 deficiency leading to brain hypothyroidism [8–11].

Unfortunately, there are not many therapeutic options for these patients, and none of them improve the neurological damage. In initial attempts, since patients present low serum T4 levels, treatment with levothyroxine (LT4) in combination with the antithyroid drug propylthiouracil was tested [12, 13]. Patients have also been treated with the TH analog 3,5-diiodothyropropionic acid (DITPA), which is an agonist of the T3 nuclear receptor [14]. These treatments lowered the serum T3 levels, decreasing the peripheral hyperthyroidism. Unfortunately none of them was able to ameliorate the psychomotor state of the patients, as both T3 and T4 entry to the brain is restricted in MCT8-deficient patients and DITPA may not reach specific brain cells. Currently another TH analog, 3,3′,5-triiodothyroacetic acid (TRIAC), is being considered as an alternative treatment and a clinical trial is under way in MCT8-deficient patients [15].

The TH analog TRIAC is considered as an alternative for the treatment for MCT8 deficiency as: i) there is extensive clinical experience with TRIAC as it has been used successfully in patients with thyroid cancer [16] and with resistance to TH [17, 18]; ii) TRIAC exerts thyromimetic actions as it binds with the same affinity as T3 to the TH receptor alpha and with higher affinity to the TH receptor beta, modulating the nuclear transcription [19]; iii) TRIAC is transported into brain cells by a transporter other than MCT8 and it induces similar neuronal gene responses as T3 *in vitro* [20]; iv) high doses of TRIAC administered intraperitoneally to newborn mice are able to prevent neuronal damage in the hypothyroid brain [20, 21].

In order to assess the effects of TRIAC treatment in MCT8-deficiency, in a previous study we administered therapeutic doses of TRIAC (30 ng/g of body weight (BW) per day) to mice lacking MCT8 (*Mct8*KO) from postnatal day 21 (P21) to P30 [22] through drinking water. This treatment restored T3 levels but severely decreased T4 levels in plasma. Also, although treatment with the established therapeutic doses of TRIAC increased 3-fold the TRIAC levels in plasma, these were insufficient to increase TRIAC content in the brain and to modulate the expression of T3-regulated genes in brain cells. Most importantly, this treatment worsened the brain hypothyroidism by further decreasing T3 content. This indicated that systemic treatment of TRIAC at therapeutic, though not excessive doses, aggravates brain hypothyroidism without producing thyromimetic effects.

However, because TRIAC possesses some characteristics that could potentially benefit MCT8-deficient patients, and because a second clinical trial is on its way (https://clinicaltrials.gov/ct2/show/NCT02396459), evaluating the effects of TRIAC administration to the younger patients in order to assess whether the neurological phenotype can be rescued, it is essential to further investigate TRIAC as a potential treatment to improve the neurological impairments of patients. Since impaired TH transport across BBs plays a major role in the neurological pathology in MCT8 deficiency, the aim of the present study is to identify alternative delivery routes that bypass the BBs in order to administer TH analogs that can exert thyromimetic actions on TH target neural cells and palliate the neurological symptoms present in MCT8-deficient patients. Intracerebroventricular (ICV) infusion provides an alternative and safe route of administration to the CNS that circumvents the BBs by delivering drugs directly into the brain lateral ventricle [23]. By this administration route, drugs can be delivered into mouse cerebrospinal fluid (CSF), allowing a widespread distribution of the drug throughout the whole ventricular system, and bypassing the BBs [24]. Furthermore, ICV delivery route is already being used in humans for the treatment of brain tumors by implantation of programmable infusion pumps [25].

Here we found that ICV administration of therapeutic doses of TRIAC to *Mct8*KO animals increases plasma TRIAC levels but does not alter plasma thyroid function tests and does not worsen brain hypothyroidism. Most importantly we have found an increase of TRIAC content in the cerebral cortex, although it was not enough to modulate the expression of T3-regulated genes in this brain region. These findings indicate that the ICV delivery route facilitates the brain availability of TRIAC.

## Materials and methods

### Animal models and experimental design

All experimental procedures involving animals were performed following the European Union Council guidelines (directive 2010/63/UE) and Spanish regulations (R.D. 53/2013) and were approved by the ethics committee at Consejo Superior de Investigaciones Científicas (CSIC; approval number 162/17).

All mice were housed at the Instituto de Investigaciones Biomédicas “Alberto Sols” under temperature- and light-controlled conditions at 22 ± 2°C on a 12:12 light–dark cycle with *ad libitum* access to food and water. Experiments were carried out in Wild type (Wt) and MCT8-deficient (*Mct8*KO) male mice. Wt and *Mct8*KO mice were originally generated by Dumitrescu and colleagues [26] and a colony was established at our animal facility in the same C57BL/6J genetic background. For the experiments Wt and *Mct8*KO littermates were obtained by backcrossing *Mct8*^−/+^ females with *Mct8*^+/y^ males. The *Mct8* genotype was confirmed by PCR of tail DNA as described [27].

Surgical implantation of osmotic minipumps into the right lateral ventricle was performed as described [24]. In brief, 3-month-old animals were anesthetized with ketamine (75 μg/g of body weight; BW) and medetomidine hydrochloride (1 μg/g of BW) and all efforts were made to minimize suffering. Mice were shaved above the skull, placed on the stereotaxic apparatus and an incision was made at the midline to expose the skull and the neck. A hole was drilled through the skull, above the right lateral ventricle (bregma–0.5 mm, 1.0 mm lateral). Next, an Alzet Brain Infusion Kit 3 (Alzet, 0008851) catheter connected to a 2002 Alzet osmotic minipump (Alzet, 0000296) was implanted at a depth of 2 mm into the lateral ventricle of 3-month-old Wt and *Mct8*KO male mice. This system allows a continuous release at a ratio of 0.5 μl per hour. Wt animals were treated with artificial cerebrospinal fluid (aCSF or vehicle treated) (n = 6) and *Mct8*KO animals treated with aCSF (n = 7) or with therapeutic doses of TRIAC (30 ng/g BW per day (n = 9). After surgery mice received 100 ng/g BW of buprenorphine to alleviate pain and they were supervised every day after surgery. Those who presented signs of pain were immediately sacrificed to prevent animal suffering. 12 days after surgery, mice were anesthetized with ketamine (75 μg/g of BW) and medetomidine hydrochloride (1 μg/g of BW) and transcardially perfused with saline to remove blood from tissues before their collection. Prior to perfusion, blood was extracted by retroorbital collection and used for the determination of T4, T3 and TRIAC concentrations in plasma. Both cerebral hemi-cortices were harvested, one for T4, T3 and TRIAC content determinations and another for RNA extraction and gene expression analysis.

### Radioimmunoassays of T4, T3, and TRIAC in plasma and tissues

High specific activity ^125^I-T3, ^125^I-T4 and ^125^I-TRIAC (3000 μCi/μg) were labelled with ^125^I (Perkin Elmer, NEZ033A) using (3–5)-T2 (Sigma, D0629), T3 (Sigma, T2877) and DIAC (Sigma, D7932) respectively as substrates as described [28, 29], only the separation of the labelled products was done by ascending paper chromatography for 16 h, in presence of Butanol:Ethanol:Ammonia 0.5N (5:1:2) as solvent. The ^125^I-T3, ^125^I-T4 and ^125^I –TRIAC were eluted and kept in ethanol at 4°C.

T3 and T4 were extracted from individual 80 μl aliquots of plasma with methanol (1:6), evaporated to dryness and taken up in the radioimmunoassay (RIA) buffer for determinations. T3 and T4 content from hemi-cortices were determined by RIA after extraction and purification as previously described [30, 31] by using methanol-chloroform extraction, back extraction into an aqueous phase, and purification of the extracts on DOWEX AG 1-X2 columns (Bio Rad, 140–1251). This procedure retains TRIAC in the DOWEX, avoiding any interference of TRIAC in the T3 RIA. The purified extracts were used for T3 and T4 determinations by sensitive RIAs [30, 31] with the dynamic range being 0.4–50 pg T3/tube and 2.5–320 pg T4/tube. Samples were processed in duplicates and the final results were calculated by using recovery data obtained by adding tracer amounts of ^125^I-T3, ^125^I-T4 to the initial homogenates. For plasma samples, T3 data were corrected for the crossreactivity of TRIAC on the T3 antiserum (17%).

TRIAC plasma levels and content from hemi-cortices were determined by RIA after extraction as previously described [32] by using methanol (1:6) extraction, evaporated to dryness and reconstituted with RIA buffer. The purified extracts were used for TRIAC determinations by sensitive RIAs [32] using a specific TRIAC antibody (kindly provided by Dr. A. Burger) with the dynamic range being 0.9 to 250 pg TRIAC/tube. Samples were processed in duplicates and the final results were calculated by using recovery data obtained by adding tracer amounts of ^125^I-TRIAC to the initial homogenates.

### Radioimmunoassays of TSH in plasma

TSH level were measured in Samuel Refetoff´s laboratory. Briefly, TSH was measured in 50 μl serum using a sensitive, heterologous, disequilibrium, double-antibody precipitation RIA [33].

### Gene expression

RNA was extracted from individual hemi-cortices using TRIZOL reagent (Invitrogen; 15596026) following the manufacturer’s recommendations with an additional chloroform extraction. RNA quality was assessed with the Agilent 2100 Bioanalyzer. For the cDNA synthesis, 250 ng of RNA was used with the high-capacity cDNA reverse transcription kit (Applied Biosystems). An aliquot of cDNA equivalent to 5 ng of RNA was used in triplicates for the Real-time PCR. The PCR program consisted of a hot start of 95°C for 10 minutes, 40 cycles of 15 seconds at 95°C and 1 minute at 60°C. It was performed using the TaqMan universal PCR master mix, No Amp Erase UNG (Applied Biosystems), on a 7900HT fast real-time PCR system (Applied Biosystems). The 18S gene was used as internal standard. For analysis, the cycle threshold was used.

The expression of the following T3-dependent genes was evaluated using Applied Biosystems TaqMan probes in the liver: *Dio1*, type 1 iodothyronine deiodinase; *Gsta2*, glutathione S-transferase, alpha 2; *Ucp2*, uncoupling protein 2; in the heart: *Hcn2*, hyperpolarization-activated cyclic nucleotide-gated channel; *Atp2a2*, sarcoplasmic reticulum Ca2+ ATPase pump; *Myh6*, myosin heavy chain, cardiac muscle alpha isoform; *Myh7*, myosin heavy chain, cardiac muscle beta isoform mRNA; and in the cerebral cortex: *Hr*, hairless; *Cbr2*, carbonyl reductase; *Flywch2*, FLYWCH family member 2; *Dio3*, type 3 iodothyronine deiodinase, *Abcd2*, ATP-binding cassette, sub-family D (ALD), member 2 and *Aldh1a1*, aldehyde dehydrogenase family 1, subfamily A1.

### Statistics

Data are expressed in scatter plots as mean ± SEM. Chi-Square tests were performed to detect outliers, which were excluded from further analyses. Differences between means were obtained by one-way analysis of variance (ANOVA) and the Bonferroni’s post hoc test to correct for multiple comparisons for parametric data and by Kruskal-Wallis test for non-parametric data. Significant differences are represented as *p<0.05, **p<0.01 and ***p<0.001. All analyses were conducted using GraphPad Prism 5.

## Results and discussion

### Effects of TRIAC treatment by ICV on plasma hormone levels and peripheral tissues

**Fig 1:**
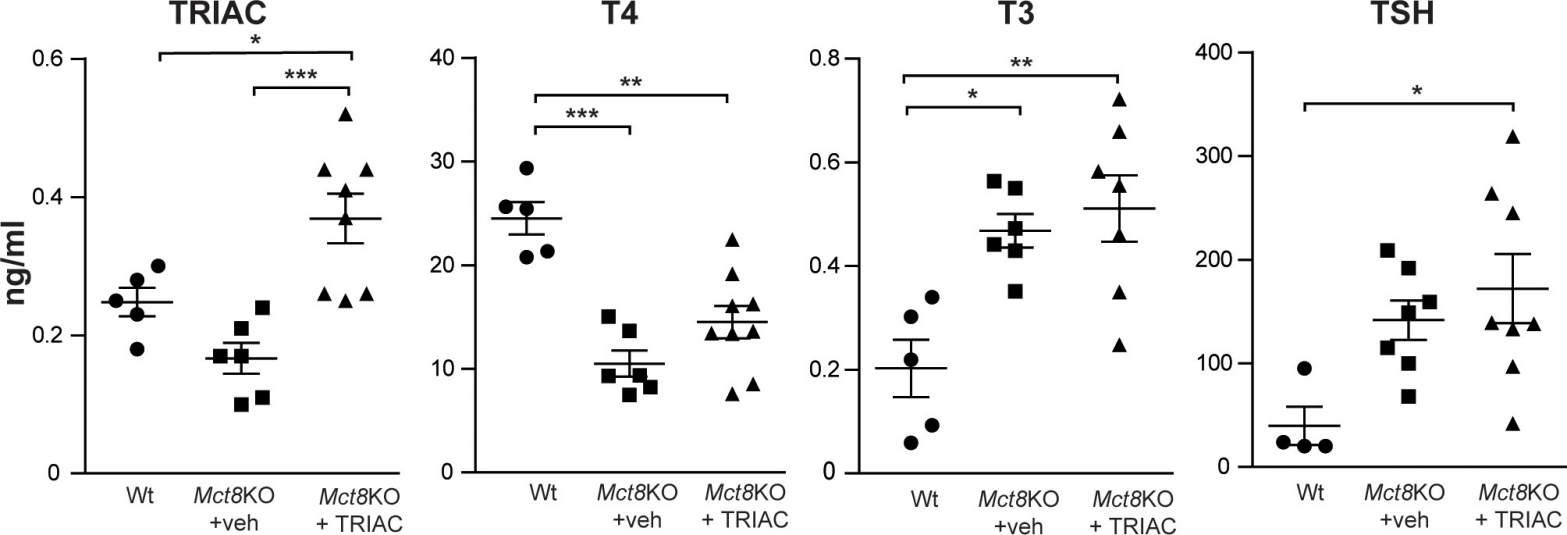
Triiodothyroacetic acid (TRIAC), thyroxine (T4) and 3,5,3’-triiodothyronine (T3) levels in plasma of vehicle treated wild type (Wt; TRIAC n = 5; T4 n = 5; T3 n = 5 and TSH n = 4) and monocarboxylate transporter 8 knock out (*Mct8*KO+veh; TRIAC n = 6; T4 n = 6; T3 n = 7 and TSH n = 7) and in *Mct8*KO mice after intracerebroventricular (ICV) administration of therapeutics doses of 30 ng/g of body weight (BW) of TRIAC per day for 12 days (*Mct8*KO+TRIAC; TRIAC n = 8; T4 n = 9; T3 n = 7 and TSH n = 8). Measures were obtained by specific radioimmunoassays. Data are expressed as scatter plots and mean ± SEM and *p<0.05, **p<0.01 and ***p<0.001 were determined by one-way analysis of variance (ANOVA) and Bonferroni’s post hoc test except for TSH levels that were determined by the Kruskal-Wallis test.

ICV administration of 30 ng of TRIAC per g body weight for 12 days caused a 2.2-fold increase in plasma TRIAC levels compared to their vehicle treated *Mct8*KO controls (Fig 1). Systemic administration of the same dose of TRIAC in the drinking water caused a 3-fold increase in plasma TRIAC levels compared with the *Mct8*KO [22]. In contrast to our previously reported systemic administration of TRIAC where the same doses of TRIAC in the drinking water caused plasma T4 levels to be 3.5-fold lower in *Mct8*KO mice than in the Wt [22], ICV treatment did not change the plasma T4 levels in the treated *Mct8*KO animals (Fig 1). As expected [26, 34], plasma T3 levels were significantly higher (1.8-fold) in vehicle treated *Mct8*KO compared with Wt animals. After ICV administration of TRIAC, T3 levels did not change in the treated *Mct8*KO animals in comparison to their vehicle treated controls (Fig 1). This finding also contrasts with our previous results, where TRIAC treatment in the drinking water decreased plasma T3 levels of *Mct8*KO animals to values similar to Wt animals [22].

Taking into account that TRIAC is a potent TSH inhibitor [35–37] and consequently can decrease plasma T4 levels [32], we measured plasma TSH. Levels in vehicle treated *Mct8*KO mice were slightly increased (not statistically significant) compared with their Wt littermates as reported [26]. Most importantly, ICV administration of TRIAC did not alter plasma TSH levels in *Mct8*KO animals after treatment (Fig 1). This finding indicates that the route of TRIAC treatment does not inhibit TSH, preventing reduction of plasma T4.

**Fig 2:**
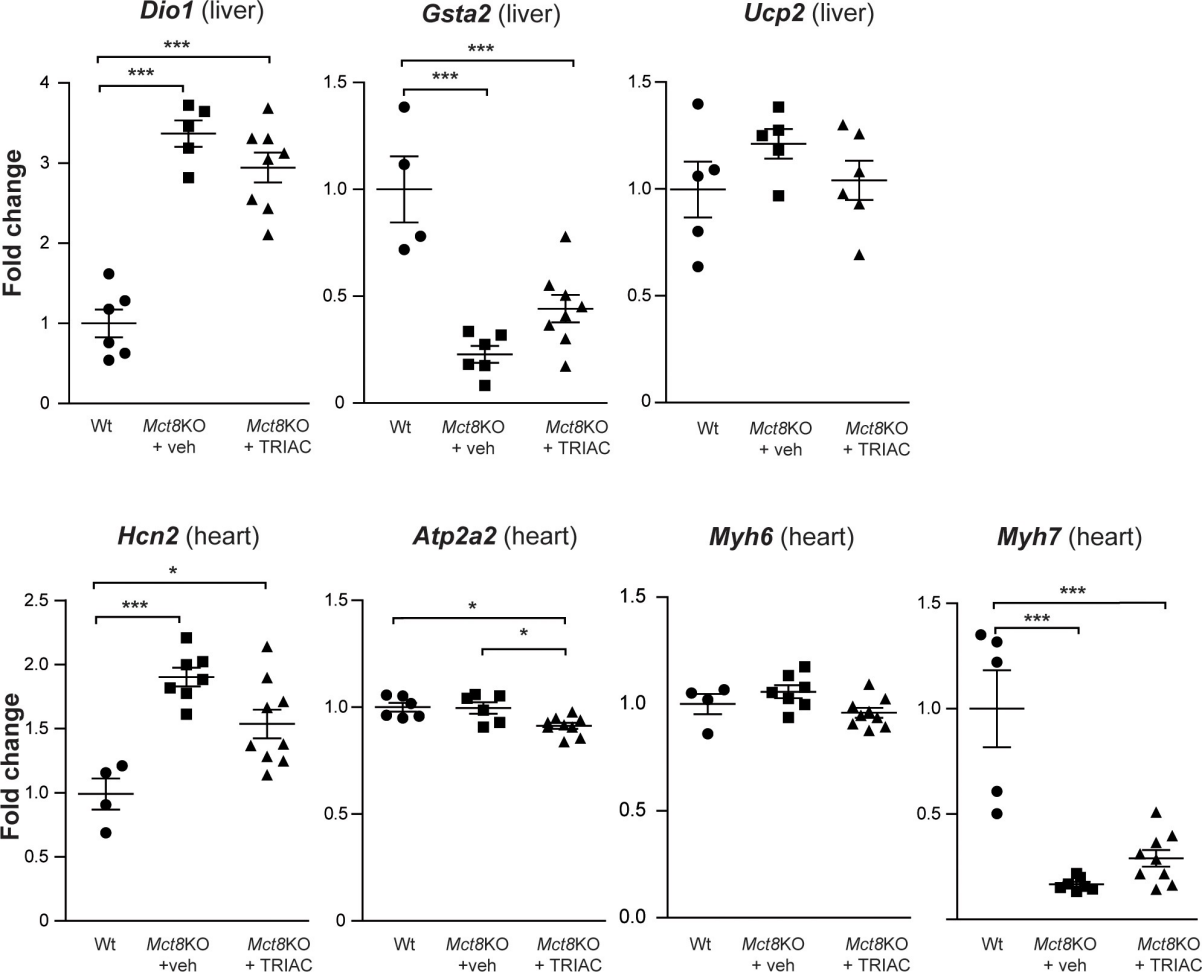
Gene expression analysis of T3-regulated genes *Dio1*, *Gsta2* and *Ucp2* in the liver and *Hcn2*, *Atp2a2*, *Myh6 and Myh7* in heart of vehicle treated Wt (*Dio1* n = 6; *Gsta2* n = 4; *Ucp2* n = 5; *Hcn2* n = 4; *Atp2a2* n = 6; *Myh6* n = 4 and *Myh7* n = 5), and *Mct8*KO mice (*Mct8*KO+veh; *Dio1* n = 5; *Gsta2* n = 6; *Ucp2* n = 5; *Hcn2* n = 7; *Atp2a2* n = 6; *Myh6* n = 7 and *Myh7* n = 7) and in *Mct8*KO mice after TRIAC treatment (*Mct8*KO+TRIAC; *Dio1* n = 8; *Gsta2* n = 8; *Ucp2* n = 6; *Hcn2* n = 9; *Atp2a2* n = 9; *Myh6* n = 9 and *Myh7* n = 9). Measurements were obtained by qPCR, and results were corrected for 18S RNA content. Data are expressed as scatter plots and mean ± SEM and *p<0.05 and ***p<0.001 were determined by one-way ANOVA and Bonferroni’s post hoc test. *Dio1*, type 1 iodothyronine deiodinase mRNA; *Gsta2*, glutathione S-transferase, alpha 2 mRNA; *Ucp2*, uncoupling protein 2 mRNA; *Hcn2*, hyperpolarization-activated cyclic nucleotide-gated channel mRNA; *Atp2a2*, sarcoplasmic reticulum Ca^2+^ ATPase pump mRNA; *Myh6*, myosin heavy chain, cardiac muscle alpha isoform mRNA and *Myh7*, myosin heavy chain, cardiac muscle beta isoform mRNA.

To study the effects of the present treatment on peripheral organs, we assessed the expression of the T3-regulated genes *Dio1*, *Gsta2* and *Ucp2* in the liver and *Hcn2*, *Atp2a2*, *Myh6 and Myh7* in the heart. In the liver, *Dio1* expression increased 3-fold, while the expression of *Gsta2* (a gene that is negatively regulated by T3) decreased more than 4-fold. *Ucp2* expression was not affected in *Mct8*KO mice compared to Wt animals. The expression levels of these genes did not return to Wt values after treatment (Fig 2). In the heart, *Hcn2* expression increased 2-fold, while *Atp2a2* was not affected in *Mct8*KO mice in comparison to the Wt. *Myh6* was also unaltered in *Mct8*KO mice, however, the expression of the beta isoform, *Myh7*, which is negatively regulated by T3, decreased 10-fold, and continued diminished after TRIAC treatment. Even though ICV treatment with TRIAC did not decrease plasma T3 levels, the expression of *Hcn2*, *Atp2a2* and *Myh6* in the heart seems to slightly decrease after treatment (Fig 2; only statistically significant for *Atp2a2*). Altogether these data suggest that ICV treatment with TRIAC does not aggravate the hyperthyroidism in peripheral tissues.

In conclusion, ICV administration of TRIAC to *Mct8*KO animals increases the plasma levels of TRIAC, without a decrease in T4 or T3 plasma levels. Therefore, although ICV delivery of therapeutic doses of TRIAC for 12 days does not improve the peripheral tissue hyperthyroidism present in *Mct8*KO animals, it does not further decrease T4 levels which could lead to detrimental effects, particularly in the CNS.

### Effects of TRIAC treatment by ICV in the CNS

**Fig 3:**
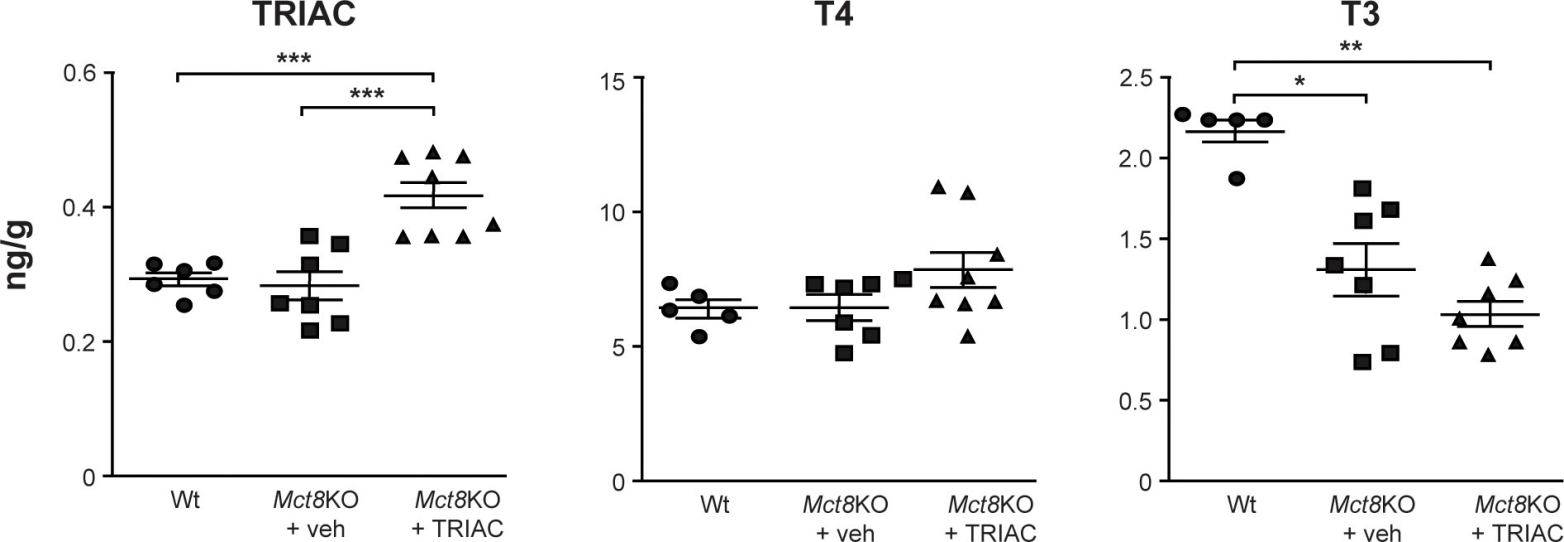
TRIAC, T4 and T3 content in the cerebral cortex in vehicle treated Wt (TRIAC n = 6; T4 n = 5 and T3 n = 5) and *Mct8*KO mice (*Mct8*KO+veh; TRIAC n = 7; T4 n = 7 and T3 n = 7) and in *Mct8*KO mice after TRIAC treatment (*Mct8*KO+TRIAC; TRIAC n = 8; T4 n = 8 and T3 n = 7). Measurements were obtained by specific radioimmunoassays. Data are expressed as scatter plots and mean ± SEM and ***p<0.001 was determined by one-way ANOVA and Bonferroni’s post hoc test for TRIAC and T4 content and **p<0.01 and *p<0.05 by the Kruskal-Wallis test for T3 content.

In order to explore the effects TRIAC treatment by ICV on CNS, the thyroidal status was assessed by determining the T4 and T3 content in the cerebral cortex. T4 content was similar in vehicle treated *Mct8*KO and Wt animals and, most importantly, TRIAC treatment by ICV did not alter T4 content in the cerebral cortex of *Mct8*KO mice (Fig 3). As already reported [26, 34] T3 was lower in the cerebral cortex of vehicle treated *Mct8*KO than the corresponding Wt mice (1.6-fold) but was not further reduced by TRIAC given by ICV (Fig 3), therefore not aggravating brain hypothyroidism. This differs from our previous studies [22], where systemic administration of the same dose of TRIAC in the drinking water decreased T3 content in the cerebral cortex and the striatum, worsening the brain hypothyroidism of *Mct8*KO animals. In the present study, as TRIAC administration by ICV did not further decrease plasma T4 levels, the detrimental effect of systemic treatment with TRIAC did not take place.

The ability of TRIAC to reach the brain when given ICV was evaluated by directly measuring the content of TRIAC in the cerebral cortex. ICV treatment of TRIAC in *Mct8*KO animals increased TRIAC content in the cerebral cortex by 1.5-fold (Fig 3). This finding indicates a clear advantage in comparison to administration of the same dose of TRIAC in the drinking water, where TRIAC content did not increase in the cerebral cortex or the striatum after treatment [22].

**Fig 4:**
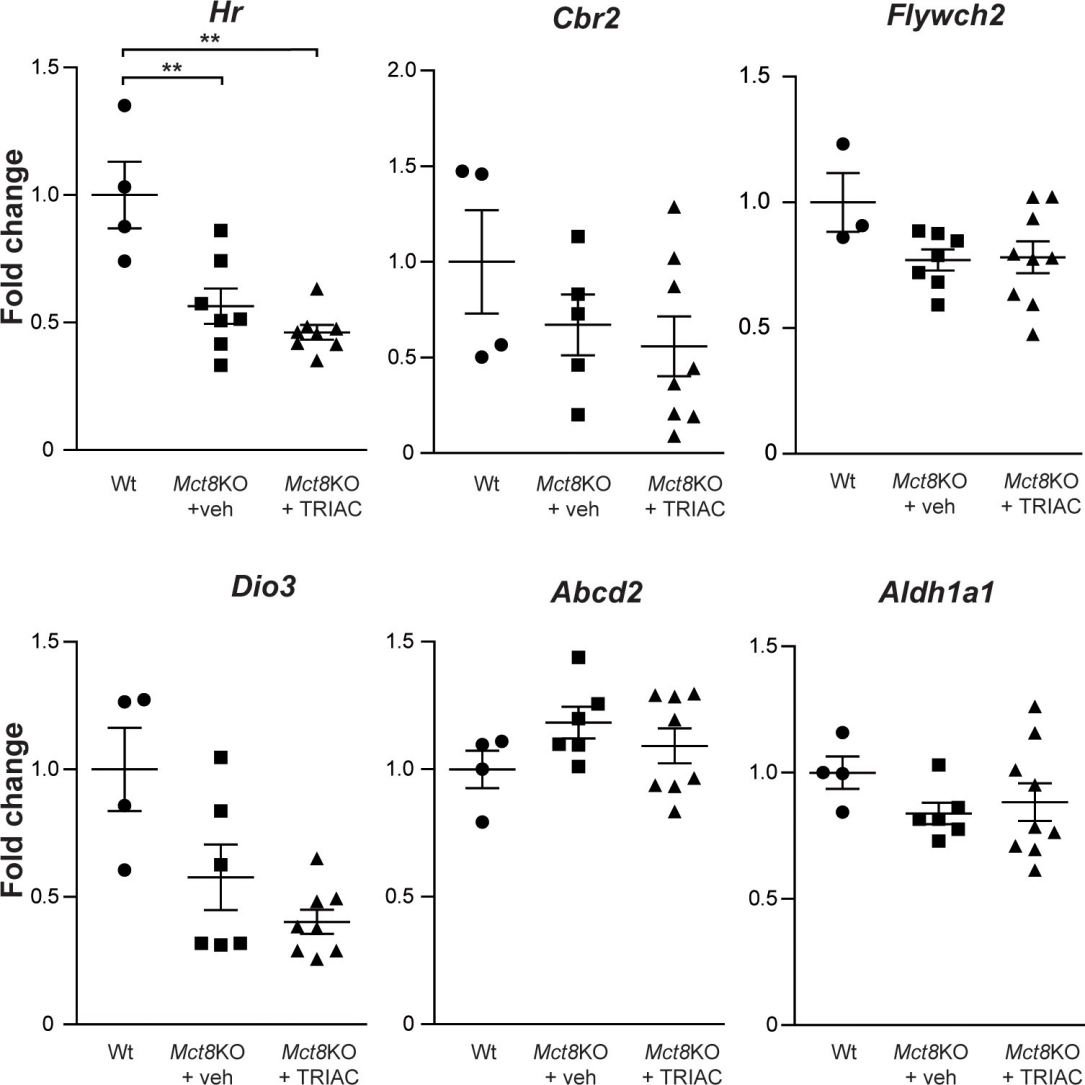
Gene expression analysis of T3-regulated genes in the cerebral cortex of vehicle treated Wt (*Hr* n = 4; *Cbr2* n = 4; *Flywch2* n = 3; *Dio3* n = 4; *Abcd2* n = 4 and *Aldh1a1 n = 4*), and *Mct8*KO mice (*Mct8*KO+veh; *Hr* n = 7; *Cbr2* n = 5; *Flywch2* n = 7; *Dio3* n = 6; *Abcd2* n = 6 and *Aldh1a1* n = 6) and in *Mct8*KO mice after TRIAC treatment (*Mct8*KO+TRIAC; *Hr* n = 8; *Cbr2* n = 8; *Flywch2* n = 9; *Dio3* n = 8; *Abcd2* n = 8 and *Aldh1a1* n = 9). Measurements were obtained by qPCR, and results were corrected for 18S RNA content. Data are expressed as scatter plots and mean ± SEM and **p<0.01 was determined by one-way ANOVA and Bonferroni’s post hoc test. *Hr*, hairless mRNA; *Cbr2*, carbonyl reductase mRNA; *Flywch2*, FLYWCH family member 2 mRNA; *Dio3*, type 3 iodothyronine deiodinase mRNA; *Abcd2*, ATP-binding cassette, sub-family D (ALD), member 2 mRNA and *Aldh1a1*, aldehyde dehydrogenase family 1, subfamily A1 mRNA.

Because the ultimate effect of TH is the regulation of gene expression, analysis of the expression of T3-regulated genes can be used to monitor the thyroidal status of different tissues and the effects of TH analogs. In order to assess if the increased TRIAC content in the brain, as a result of ICV treatment, has an effect on T3-regulated genes, we studied the expression of a set of T3-dependent genes in the cerebral cortex. We analyzed *Hr* and *Cbr2*, two genes whose expression is diminished in *Mct8*KO [27] in comparison to Wt mice, and of *Flywch2*, *Dio3*, *Aldh1a1* and *Abcd2* which are known T3-responsive genes [38] in the cerebral cortex. Despite the increase in the brain TRIAC content after ICV administration it did not stimulate the expression of any of the T3-responsive genes studied (Fig 4).

In conclusion, ICV administration of therapeutic doses of TRIAC, increased TRIAC content in the cerebral cortex, without reducing endogenous T3 and T4. However, it did not affect the expression of the T3-regulated genes. Nevertheless, it does not worsen the brain hypothyroidism of *Mct8*KO animals as observed in animals given TRIAC systemically.

The lack of effect of TRIAC treatment by ICV on the expression of T3-regulated genes could be due to different aspects. The first one is the hormone sensitivity of *Mct8*KO mice as these mice present the peripheral alterations characteristic of patients, however, they do not present gross neurological abnormalities. In contrast to humans, mice abundantly express an alternative TH transporter, the organic anion transporting polypeptide 1c1 (OATP1C1), at the BBs that allows T4 to enter the brain. This T4, through the action of DIO2, provides enough T3 to maintain some functions, such as the regulation of most T3-regulated genes. The use of an alternative mice model with lower brain T3 content and similar neurological alterations as in patients, such as the double mutants *Mct8/Dio2KO* [39] or *Mct8/Oatp1c1KO* [40], could provide more sensitive results.

The dose of TRIAC, the duration and timing of the treatment would also require further investigation. *In vitro* studies have demonstrated that TRIAC has the ability to induce the expression of T3-regulated genes and, *in vivo*, administration of high doses (200–400 ng/g BW per day) of TRIAC to *Mct8/Oatp1c1KO* at very early stages of development has shown to prevent neuronal damage [21]. However, in our present work, ICV administration of 30 ng/g BW per day only increased TRIAC content in the brain in 0.1 ng per gram of tissue weight, demonstrating the viability of the intracerebral injection but suggesting that it might not be enough to induce gene expression changes. Moreover, the increase of TRIAC levels in serum after ICV administration, did not promote the expression of T3-target genes in peripheral organs questioning whether TRIAC serves as a TH analog exerting T3-like effects. The use of higher doses of TRIAC for a longer period at earlier stages of development might shed light into this matter.

Finally, it is important to mention the possible implications of TRIAC transport into and out of cells. The fact that TRIAC administered in CSF can reach the blood, however, when administered systemically does not reach the brain, could be indicating a putative TRIAC transporter that can transport TRIAC from the BBs into the plasma but not the other way. Moreover, the binding of TRIAC to transporter proteins in CSF such as transthyretin that allow TRIAC transfer into the brain across the blood-cerebrospinal fluid barrier should also be considered [41].

## Conclusions

Our previous findings in *Mct8*KO mice indicate that systemic administration of therapeutic doses of TRIAC do not reach the brain and even aggravate brain hypothyroidism [22]. In the present study we found that administration of the same dose of TRIAC directly into the brain by ICV does not aggravate the hyperthyroidism in peripheral tissues, nor further decreases plasma T4 levels. Thus, this mode of therapy does not aggravate brain hypothyroidism. Most importantly, ICV treatment increases TRIAC content in the brain, although under the present conditions it did not modulate the expression of the studied T3-regulated genes. The data suggest that ICV delivery of TRIAC presents advantages over systemic administration to target the brain and ultimately address the neurological impairments of MCT8-deficient patients.
